# Notification that new names of prokaryotes, new combinations and new taxonomic opinions have appeared in volume 74, part 10 of the IJSEM

**DOI:** 10.1099/ijsem.0.006588

**Published:** 2025-01-31

**Authors:** Aharon Oren, Markus Göker

**Affiliations:** 1 The Institute of Life Sciences, The Hebrew University of Jerusalem, The Edmond J. Safra Campus, 9190401 Jerusalem, Israel; 2 Leibniz Institute DSMZ – German Collection of Microorganisms and Cell Cultures, Inhoffenstrasse 7B, 38124 Braunschweig, Germany

This listing of names of prokaryotes published in a previous issue of the *IJSEM* is provided as a service to microbiology to assist in the recognition of new names and new combinations. This procedure was proposed by the Judicial Commission [1]. The names given herein are listed according to the Rules of priority (i.e. time and date of publication of the articles on the journal’s platform and the order of valid publication of names in the original articles) [2].

**Table:** 

Order of article publication	Name/authors	Proposed as	Article no.	Page no.
1	*Adlercreutzia wanghongyangiae* Liu *et al*. 2024	sp. nov.	006531	6
1	*Adlercreutzia shanghongiae* Liu *et al*. 2024	sp. nov.	006531	6
2	*Rhodococcus navarretei* Carrasco *et al*. 2024	sp. nov.	006536	9
2	*Pseudarthrobacter quantipunctorum* Carrasco *et al*. 2024	sp. nov.	006536	11
3	*Halostreptopolyspora* Li *et al*. 2024	gen. nov.	006484	7
3	*Halostreptopolyspora alba* Li *et al*. 2024	sp. nov.	006484	7
4	*Rhodobacter flavimaris* Huang *et al*. 2024	sp. nov.	006540	8
4	*Rhodobacter flavescens* (Mu *et al*. 2024) Huang *et al*. 2024	comb. nov. [basonym: *Sedimentimonas flavescens* Mu *et al*. 2024]	006540	8
4	*Rhodobacter enshiensis* (Wang *et al*. 2014) Huang *et al*. 2024	comb. nov. [basonym: *Paenirhodobacter enshiensis* Wang *et al*. 2014]	006540	8
4	*Rhodobacter ferrireducens* (Yang *et al*. 2018) Huang *et al*. 2024	comb. nov. [basonym: *Sinirhodobacter ferrireducens* corrig. Yang *et al*. 2018]	006540	8
4	*Rhodobacter hankyongi* (Lee *et al*. 2020) Huang *et al*. 2024	comb. nov. [basonym: *Sinirhodobacter hankyongi* Lee *et al*. 2020]	006540	9
4	*Rhodobacter huangdaonensis* (Xi *et al*. 2019) Huang *et al*. 2024	comb. nov. [basonym: *Sinirhodobacter huangdaonensis* corrig. Xi *et al*. 2019]	006540	9
4	*Rhodobacter populi* (Xu *et al*. 2019) Huang *et al*. 2024	comb. nov. [basonym: *Sinirhodobacter populi* corrig. Xu *et al*. 2019]	006540	9
5	*Campylobacter californiensis* Miller *et al*. 2024	sp. nov.	006524	9
6	*Allocatenococcus* Oren and Molinari Novoa 2024	gen. nov.	006547	1
6	*Allocatenococcus thiocycli* (Sorokin 1994) Oren and Molinari Novoa 2024	comb. nov. [basonym: *Catenococcus thiocycli* corrig. Sorokin 1994]	006547	2
6	*Allocoenonia* Oren and Molinari Novoa 2024	gen. nov.	006547	2
6	*Allocoenonia anatina* (Vandamme *et al*. 1999) Oren and Molinari Novoa 2024	comb. nov. [basonym: *Coenonia anatina* Vandamme *et al*. 1999]*	006547	2
6	*Allofournierella* Oren and Molinari Novoa 2024	gen. nov.	006547	3
6	*Allofournierella massiliensis* (Togo *et al*. 2017) Oren and Molinari Novoa 2024	comb. nov. [basonym: *Fournierella massiliensis* Togo *et al*. 2017]	006547	3
7	*Polymorphospora lycopeni* Liu *et al*. 2024†	sp. nov.	006543	8
8	*Paenibacillus suaedae* Kim *et al*. 2024	sp. nov.	006542	6
8	*Paenibacillus violae* Kim *et al*. 2024	sp. nov.	006542	7
9	*Thioclava arctica* Pan *et al*. 2024	sp. nov.	006548	10
9	*Zhongshania arctica* Pan *et al*. 2024	sp. nov.	006548	12
10	*Yoonia algicola* Han *et al*. 2024	sp. nov.	006545	8
10	*Yoonia rhodophyticola* Han *et al*. 2024	sp. nov.	006545	8
10	*Yoonia phaeophyticola* Han *et al*. 2024	sp. nov.	006545	9
11	*Sphingomonas rustica* Lee *et al*. 2024‡	sp. nov.	006551	7
11	*Sphingomonas agrestis* Lee *et al*. 2024§	sp. nov.	006551	8
12	*Leuconostoc koreense* Lee *et al*. 2024	sp. nov.	006533	8
13	*Sorlinia* Marasco *et al*. 2024	gen. nov.	006544	11
13	*Sorlinia euscelidii* Marasco *et al*. 2024¶	sp. nov.	006544	11
14	*Hydrogenimonas leucolamina* Hatakeyama *et al*. 2024	sp. nov.	006553	11
15	*Flavobacterium capsici* Cho *et al*. 2024#	sp. nov.	006554	8
16	*Bifidobacterium fermentum* Breselge *et al*. 2024	sp. nov.	006549	8
16	*Bifidobacterium aquikefiricola* Breselge *et al*. 2024	sp. nov.	006549	9
17	*Janibacter alittae* Kim *et al*. 2024	sp. nov.	006557	5
18	*Paracoccus actinidiae* Xu *et al*. 2024	sp. nov.	006529	10
19	*Fidelibacter* Katayama *et al*. 2024	gen. nov.	006558	6
19	*Fidelibacter multiformis* Katayama *et al*. 2024**	sp. nov.	006558	6
19	*Fidelibacteraceae* Katayama *et al*. 2024	fam. nov.	006558	6
19	*Fidelibacterales* Katayama *et al*. 2024	ord. nov.	006558	7
19	*Fidelibacteria* Katayama *et al*. 2024	class. nov.	006558	7
19	*Fidelibacterota* Katayama *et al*. 2024	phyl. nov.	006558	7
20	*Sodalis praecaptivus* subsp. *praecaptivus* (Chari *et al*. 2015) Teh *et al*. 2024††	subsp. nov.	006552	9
20	*Sodalis praecaptivus* subsp. *spalangiae* Teh *et al*. 2024‡‡	subsp. nov.	006552	9
21	*Amycolatopsis melonis* Xu *et al*. 2024	sp. nov.	006559	7
22	*Paracoccus spongiarum* Kim *et al*. 2024	sp. nov.	006566	7

*The basonym has a risk group of 2 (German TRBA risk group classification, accessed 29 October 2023).

†The protologue heading must read *Polymorphospora lycopeni* instead of *P. lycopeni*.

‡The protologue heading must read *Sphingomonas rustica* instead of *S. rustica*.

§The protologue heading must read *Sphingomonas agrestis* instead of *S. agrestis*.

¶The protologue heading must read *Sorlinia euscelidii* instead of *S. euscelidii*.

#The protologue heading must read *Flavobacterium capsici* instead of *F. capsici*.

**The protologue heading must read *Fidelibacter multiformis* instead of *F. multiformis*.

††The protologue heading must read *Sodalis praecaptivus* subsp. *praecaptivus* instead of *S. praecaptivus* subsp. *praecaptivus*.

‡‡The protologue heading must read *Sodalis praecaptivus* subsp. *spalangiae* instead of *S. praecaptivus* subsp. *spalangiae*.

The following taxonomic opinions (i.e. the emendation of circumscriptions or the creation of synonyms) do not affect the status of a name under the International Code of Nomenclature of Prokaryotes. These taxonomic opinions can neither be considered approved nor disapproved by the International Committee on Systematics of Prokaryotes.

**Table:** 

Name/authors:	Article no.	Page no.
*Marortus luteolus* Yu *et al*. 2019 pro synon. *Zhongshania marina* On *et al*. 2019	006548	10
*Rhodobacter* Imhoff *et al*. 1984 emend. Huang *et al*. 2024	006540	8
*Sodalis praecaptivus* Chari *et al*. 2015 emend. Teh *et al*. 2024*	006552	8

*The protologue heading must read *Sodalis praecaptivus* instead of *S. praecaptivus*.
